# Supplementary material for: Dimeric Product of Peroxy Radical Self-Reaction Probed with VUV Photoionization Mass Spectrometry and Theoretical Calculations: The Case of C2H5OOC2H5
Source: Int J Mol Sci. 2023 Feb 13;24(4):3731. doi: 10.3390/ijms24043731 (PMC9965172; doi:10.3390/ijms24043731)
Supplement: Supplementary file 1 [file ijms-24-03731-s001.zip › ijms-2180618-supplementary.pdf]

Supplementary Materials

Dimeric product of peroxy radical self-reaction probed with VUV photoionization mass spectrometry and theoretical calculations: The case of $C_2H_5OOC_2H_5$

Hao Yue [1,2†], Cuihong Zhang [1,2,3†], Xiaoxiao Lin [1], Zuoying Wen [1], Weijun Zhang [1], Sabah Mostafa[3], Pei-Ling Luo[4], Zihao Zhang [5], Patrick Hemberger [5], Christa Fittschen [3,*] , and Xiaofeng Tang [1,*]

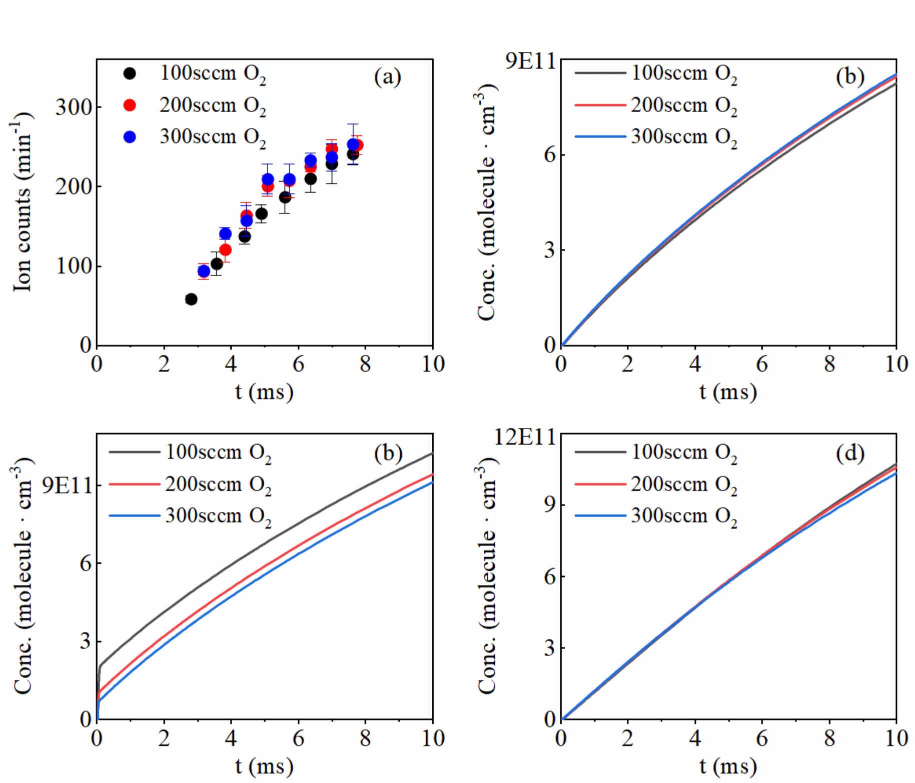

Figure S1. Time-evolutions of the dimeric product $C_2H_5OOC_2H_5$ with different flow rates of oxygen. (a) Experimental results. (b) Simulated results with the major reactions listed in Table 1. (c) Simulated results with the addition of the C_2H_5 + $C_2H_5O_2$ reaction. (d) Simulated results with the addition of the C_2H_5O + C_2H_5O reaction. The experimental results show that the ion counts of the dimeric product $C_2H_5OOC_2H_5$ increase with the concentrations of oxygen, not like the simulated cases of (c) and (d). Thus the contributions of the reactions of C_2H_5 + $C_2H_5O_2$ and C_2H_5O + C_2H_5O to the dimeric product $C_2H_5OOC_2H_5$ can be ignored.

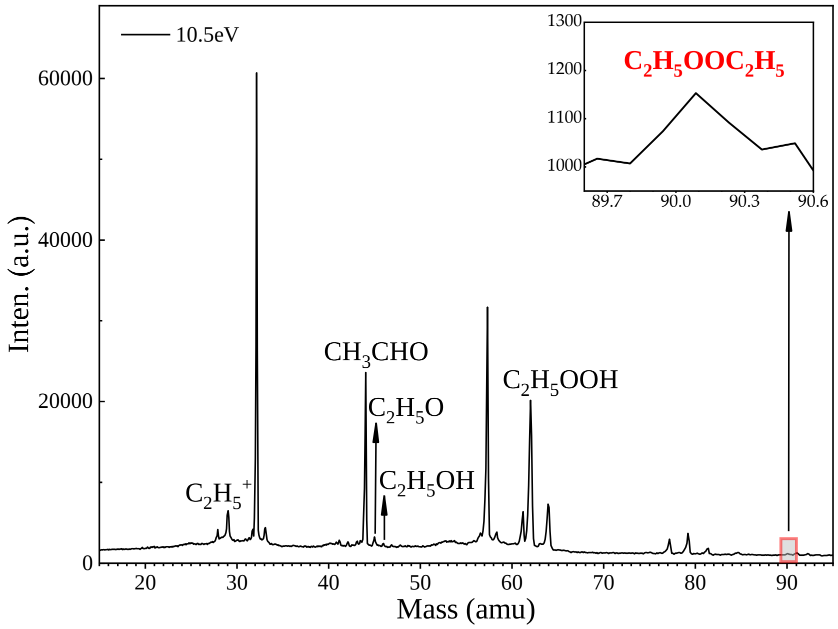

Figure S2. Synchrotron photoionization mass spectra measured at the Swiss Light Source (hν = 10.5 eV). The mass peak of $C_2H_5OOC_2H_5$ is amplified and presented in the inset.

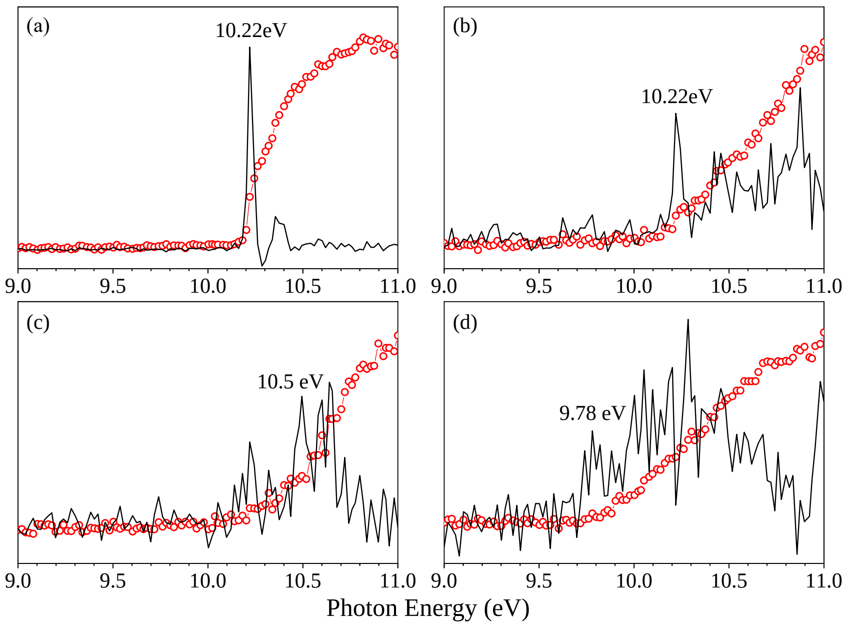

Figure S3. Mass-selected threshold photoelectron spectra (TPES, in black) and photoionization spectra (in red) of (a) CH_3CHO, m/z = 44, (b) C_2H_5O, m/z = 45, (c) C_2H_5OH, m/z = 46, and (d) C_2H_5OOH, m/z = 62.

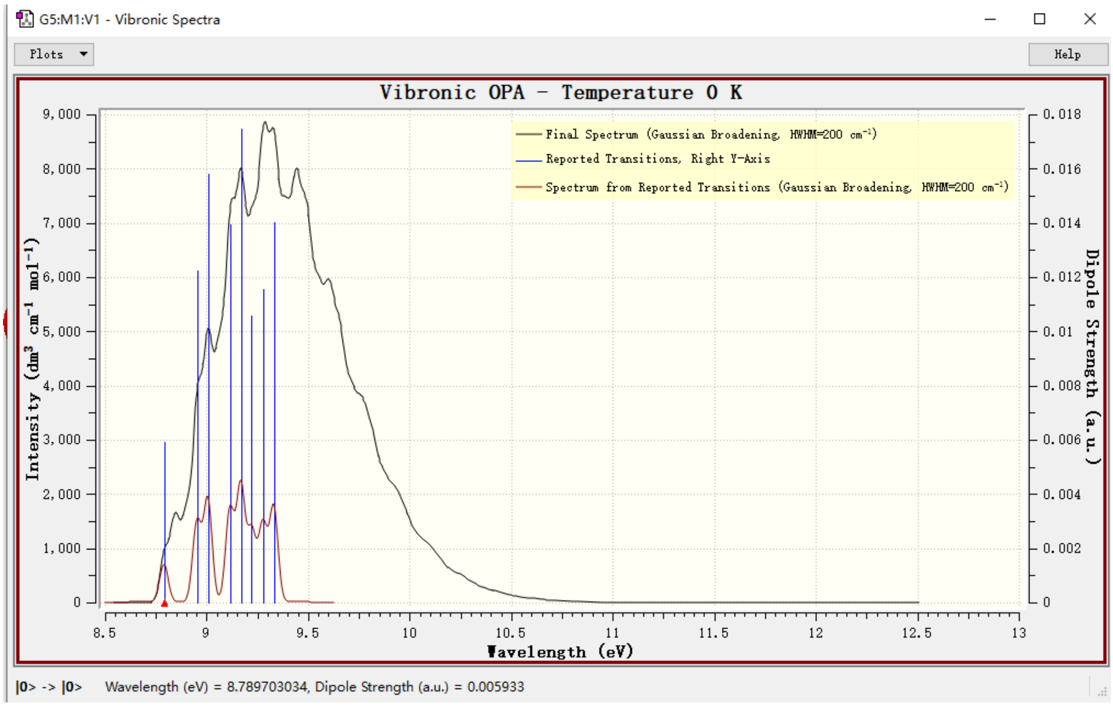

Figure S4. Calculated Franck-Condon factors and photoelectron spectrum involved in the photoionization of $C_2H_5OOC_2H_5$.

Table S1. The computed structures (xyz coordinates) and energies of key species involved in the self-reaction of $C_2H_5O_2$.

**$^2C_2H_5O$**			
Sum of electronic and zero-point Energies = -229.450098 Hartree			
	Coordinates (Angstroms)		
	X	Y	Z
O	0.63029800	-0.47389400	0.00000000
O	1.81274300	0.05767000	0.00000000
C	-0.40568600	0.53518300	0.00000000
C	-1.73214800	-0.18165400	0.00000000
H	-0.25589700	1.14708100	-0.88821200
H	-0.25589700	1.14708100	0.88821200
H	-2.54046200	0.54758000	0.00000000
H	-1.83191000	-0.80786000	-0.88465600
H	-1.83191000	-0.80786000	0.88465600
**$^1(C_2H_5O\cdots{}^3O_2\cdots OC_2H_5)$**			
Sum of electronic and zero-point Energies = -458.915504 Hartree			
	Coordinates (Angstroms)		
	X	Y	Z
O	-1.28362900	0.18644800	0.57114400
C	-2.43059900	-0.27530200	-0.14111600

C	-3.42114400	0.86600000	-0.12713200
H	-2.82368500	-1.16152200	0.35996100
H	-2.13481600	-0.53604100	-1.15813500
H	-4.32595600	0.57095100	-0.65604400
H	-3.68688800	1.13156800	0.89455800
H	-3.00120900	1.74206200	-0.61784900
O	1.28363500	0.18641500	-0.57116900
C	2.43060100	-0.27529800	0.14112300
C	3.42113300	0.86601400	0.12712300
H	2.82370500	-1.16152900	-0.35992000
H	2.13480500	-0.53601200	1.15814600
H	4.32594500	0.57098500	0.65604700
H	3.68688300	1.13156300	-0.89457000
H	3.00118600	1.74208100	0.61781800
O	-0.34024600	-0.84782800	0.60709800
O	0.34025000	-0.84785900	-0.60707300

$^3(C_2H_5O\cdots OC_2H_5)$

Sum of electronic and zero-point Energies = -308.582099 Hartree

	Coordinates (Angstroms)		
	X	Y	Z
O	1.34380000	1.36362100	0.10608500
C	1.91746300	0.24996600	-0.44666400
C	2.20667900	-0.86850800	0.53701900
H	2.80474500	0.55028500	-1.02233200
H	1.19111500	-0.09106400	-1.20647000
H	2.64926500	-1.72254600	0.02508000
H	2.89788300	-0.52850500	1.30691900
H	1.28325600	-1.19562200	1.01306700
O	-1.01551400	-1.03189700	-0.55290800
C	-1.45447700	0.15733000	-0.03857200
C	-2.93151600	0.19652600	0.30627800
H	-1.15506400	0.97344400	-0.71502900
H	-0.82398700	0.33279700	0.85267800
H	-3.19697900	1.15999800	0.74035500
H	-3.53239600	0.04401300	-0.58895600
H	-3.17302300	-0.58847400	1.02091100

$^3O_2$

Sum of electronic and zero-point Energies = -150.320798 Hartree

	Coordinates (Angstroms)		
	X	Y	Z
O	0.00000000	0.00000000	0.59491400
O	0.00000000	0.00000000	-0.59491400

^{3}TS-H-shift

One Imaginary Frequency = -923.65 cm^{-1}

Sum of electronic and zero-point Energies = -308.573077 Hartree

	Coordinates (Angstroms)		
	X	Y	Z
O	0.97179000	1.28980300	0.12038900
C	1.73767500	0.23534200	-0.36349100
C	1.80017800	-0.95176600	0.58107700
H	2.73926800	0.65589800	-0.52863600
H	1.37869100	-0.07664800	-1.35372400
H	2.46970700	-1.72152800	0.19673300
H	2.15259600	-0.63465100	1.56169500
H	0.80992600	-1.39476000	0.69483000
O	-1.12427600	-0.78842400	-1.10852200
C	-1.34539700	0.31066400	-0.37010100
C	-2.08020200	-0.00871600	0.93189000
H	-1.92510900	1.01670100	-0.98722000
H	-0.32930100	0.87072300	-0.09864500
H	-2.20991100	0.91364400	1.49343800
H	-3.05128600	-0.45399800	0.72209900
H	-1.48821400	-0.69954900	1.52824100

$^{2}C_2H_5O$

Sum of electronic and zero-point Energies = -154.289224 Hartree

	Coordinates (Angstroms)		
	X	Y	Z
C	1.18042500	-0.19736600	-0.00010400
C	-0.17529900	0.48505300	-0.00195300
O	-1.24872500	-0.36503500	-0.00298200
H	1.98313900	0.53967100	0.02045400
H	1.27641300	-0.84261700	0.87153900
H	1.29533700	-0.80824600	-0.89411800
H	-0.30628700	1.12577700	0.88649500
H	-0.28955700	1.17957700	-0.84816700

$^{1}C_2H_5OH$

Sum of electronic and zero-point Energies = -154.949438 Hartree

	Coordinates (Angstroms)		
	X	Y	Z
C	-0.08304500	0.54833300	0.00001700
H	-0.14169300	1.18964100	0.88474300
H	-0.14164700	1.18970300	-0.88466800
O	-1.14471700	-0.39433100	-0.00004300

C	1.21694200	-0.22214400	0.00002400
H	2.06619700	0.46001800	0.00007000
H	1.27725900	-0.85657400	0.88300200
H	1.27730600	-0.85651100	-0.88299700
H	-1.98307100	0.07123100	-0.00005200

$^3CH_3CHO$

Sum of electronic and zero-point Energies = -153.643986 Hartree

	Coordinates (Angstroms)		
	X	Y	Z
C	0.19992100	0.49120400	-0.13641400
H	0.36031200	1.46388400	0.33665700
O	1.22791900	-0.31178700	0.02790400
C	-1.15132000	-0.16019400	0.01817900
H	-1.30425300	-0.54231500	1.03167400
H	-1.91952900	0.57645000	-0.20262700
H	-1.25149200	-0.98978700	-0.67952500

$^1C_2H_5OOC_2H_5$

Sum of electronic and zero-point Energies = -308.633659 Hartree

	Coordinates (Angstroms)		
	X	Y	Z
O	-0.63588800	0.31685300	-0.54501600
C	-1.50079700	-0.49260500	0.23223400
C	-2.85537300	0.17949300	0.21451000
H	-1.10877700	-0.57361800	1.25039600
H	-1.54343000	-1.49271100	-0.20429300
H	-3.56672200	-0.40875300	0.79212700
H	-2.79577100	1.17659400	0.64765400
H	-3.22380900	0.26586100	-0.80622400
O	0.63588800	-0.31684200	-0.54502200
C	1.50079800	0.49260100	0.23224300
C	2.85537300	-0.17949800	0.21450700
H	1.10877700	0.57359500	1.25040700
H	1.54343200	1.49271500	-0.20426600
H	3.56672200	0.40873700	0.79213500
H	2.79577000	-1.17660700	0.64763200
H	3.22380800	-0.26584800	-0.80622900
